# The Effect of Psychological Capital and Role Conflict on the Academic Entrepreneurial Intents of Chinese Teachers in Higher Education: A Study Based on the Theory of Planned Behavior

**DOI:** 10.3389/fpsyg.2022.793408

**Published:** 2022-03-17

**Authors:** Kai Liao, Ziyang Liu, Bing Li

**Affiliations:** ^1^Business School, Hunan Institute of Technology, Hengyang, China; ^2^Department of Global Business, Kyonggi University, Suwon-si, South Korea; ^3^College of Art and Design, Shenzhen University, Shenzhen, China

**Keywords:** psychological capital, role conflict, academic entrepreneurial intents, theory of planned action, academic entrepreneurial behavior

## Abstract

Because academic entrepreneurship is an innovation driving force in China’s economy, teachers are key knowledge creators in the process of entrepreneurship. Therefore, it is particularly important to give attention to the individual psychological mechanism factors at play in the process of teachers in higher education academic entrepreneurship. The purpose of this study is to identify individual psychological capital and role conflict issues among university teachers in China. To accomplish this aim, we investigated the emergence of positive academic entrepreneurial intents, continued through the process of academic entrepreneurship, and clarified the impact of psychological capital and role conflict on entrepreneurial intent. Based on the theory of planned behavior, we constructed a research model from the perspective of entrepreneurial intent prior to entrepreneurial action. We established a cohort of teachers in 17 higher education institutions (*N* = 525) in southern China, with psychological capital and role conflict as the prior independent variables and the teachers’ academic entrepreneurial intent as the dependent variable. Using quantitative analysis, SPSS 22.0, and AMOS 23.0, we conducted reliability and validity tests, correlation analysis, and structural equation models on the collected data. We reached the following conclusions: (1) psychological capital has a positive effect on attitudes toward academic entrepreneurship; (2) psychological capital has a positive effect on perceived behavioral control; (3) role conflict has a negative effect on perceived behavioral control; (4) academic entrepreneurial attitudes have a positive effect on academic entrepreneurial intent; (5) perceived behavioral control has a positive effect on academic entrepreneurial intent; (6) subjective norms have a positive effect on academic entrepreneurial intent. We also provide some suggestions about academic entrepreneurship for university administrators.

## Introduction

Academic entrepreneurship is defined as an activity that transcends the traditional role of academic teaching or research and has innovative and risky elements that bring economic rewards to the individual academic or the institution (technology transfer and creation of spin-off companies, etc.). These rewards can be achieved by earning reputation, prestige, and social influence directly or indirectly (Abreu and Grinevich, 2013). Many scholars believe that collaborative research, contract research, consulting, and informal relationships are the achievements of university academic entrepreneurship (Perkmann et al., 2013). The case of academic startups in Italian universities reflects how governments and academic institutions have been working to create better conditions for the successful commercialization of academic research achievements (Baldini et al., 2015). A qualitative analysis was conducted the assess the entrepreneurial behavior of 30 life sciences researchers from Australian universities. All of the respondents mentioned that capital needs and institutional policy are the main driving factors of research commercialization. Academic entrepreneurial behavior is categorized into non-entrepreneurial, semi-entrepreneurial, pre-entrepreneurial, and entrepreneurial behaviors. The prerequisites for academic entrepreneurial behaviors include commercialized research opportunities, individual as opposed to commercialized team research, and appropriate funding (Holley and Watson, 2017). The traditional definition of academic entrepreneurship centers on emerging academic entrepreneurship that generates direct financial returns and provides a wider social and economic benefit to the university ecosystem (Siegel and Wright, 2015).

Many studies have identified the connections between the specific processes of academic entrepreneurship in higher education and collaboration, including connections between individuals, organizations, and institutions. However, some scholars have argued that when investigating the entrepreneurial intent of teachers in higher education, academic entrepreneurship should be viewed from the perspectives of both economic development and individual psychology.

Technology project incubation departments in eight Brazilian universities used the development of innovative and entrepreneurial universities as a theoretical basis. They found that although project incubation departments in universities preferred to work on specific academic research projects, companies created based on academic achievements were not prioritized. Meanwhile, little effort has been made to engage the academic research audience, resulting in underutilized academic research transfer channels (Stal et al., 2016). In the academic entrepreneurial process, individuals may take on more than one role. Therefore, it is important to identify all of the stakeholders that are potentially involved. Paying more attention to how researchers are rewarded and motivated and the ways that informal collaborations (consulting arrangements, joint publications, etc.) between universities and their industry partners begin is important to achieving success in academic entrepreneurship (Wood, 2011). A bibliometric analysis of the common terms and content of 615 academic ventures on the Web of Science identified four interrelated clusters: (1) components of entrepreneurial universities; (2) university spin-off activities and commercialization; (3) characteristics, motivations, and barriers of academic entrepreneurship; (4) knowledge transfer and regional economies (Skute, 2019). Although previous research has provided important insights on academic entrepreneurship, no one as of yet has explored how organizational environmental factors motivate teachers to form positive academic entrepreneurial intents. The aim of the present study is to contribute to the existing research from the perspectives of psychological capital and role conflict. It adopts the theory of planned behavior as a research framework and provides new ideas about the psychological mechanism of how academic entrepreneurial intent is formed among university teachers.

## Literature Review

### Psychological Capital

Previous studies have generally recognized that psychological capital is a core psychological element that transcends human and social capital and functions as a psychological resource for individual growth and performance enhancement (Luthans et al., 2004, 2007). Compared to human and social capital, psychological capital is a more positive predictor of subjective career success and organizational sponsorship. It can take the form of recognition by superiors, training and development opportunities, organizational resources, and information and fully mediates the relationship between psychological capital and objective career success (Zhou et al., 2015). Psychological capital is positively related to individual job performance, and it leads to a level of performance that exceeds personality self-ratings and core self-ratings (Avey et al., 2010). Effective development of psychological capital needs to be executed in the right context, and it involves training in diverse and specific skills and behavioral patterns. The development of psychological capital contributes to the formation of positive thinking patterns and can change and replace individuals’ previously ingrained beliefs and perceptions. This type of shift requires an open and positive environment in which new ideas, plans, and a sense of control are accepted (Luthans and Youssef-Morgan, 2017).

Few researchers have investigated the work motivation and psychological capital of teachers. One study found that administrators should attend to the influence of psychological capital on teachers’ positive psychology with the aim of enhancing positive work motivation and providing high-quality education (Viseu et al., 2016). We investigated the psychological capital and job satisfaction of 104 members of higher education institutions in Thailand. Correlation analysis found that there was a positive correlation between psychological capital and job satisfaction and that psychological capital and job satisfaction were negatively correlated with organizational employee turnover tendency (Salam, 2017). The perceptions that teachers hold about the authentic leadership of principals in China’s Taiwan were also studied, and it was found that this type of leadership was positively associated with teachers’ psychological capital, which shows that authentic leadership behaviors foster positive psychological capital in organizational members (Feng, 2016). Positive psychological capital can be effective in preventing and reducing depressive symptoms of employees or teachers that stem from work stress (Shen et al., 2014; Han et al., 2019). Studies in Malaysia and Egypt have shown that psychological capital is a positive predictor of entrepreneurial success (Juhdi et al., 2015; Elsafty et al., 2020). Based on these findings, this study focuses on the mechanisms that influence the psychological capital of university teachers in academic entrepreneurship.

### Role Conflict

Individuals in different social situations have different value perceptions of the roles that they play. Conflict arises when the value perceptions are inconsistent or when individuals play two or more roles (Kahn et al., 1964). Scholars found that role conflict is particularly strong among university teachers. University administrators must have a clear understanding of which university teachers are (background), what they want to be (mission), who they want to serve (trajectory), and how the university finds effective management strategies so that existing role conflicts within the university can be reduced (Wolverton et al., 1999). Burnout as a teacher-researcher role conflict was investigated, and it was found that role conflict was positively associated with teachers’ emotional exhaustion and that organizational support could mitigate the reduced personal fulfillment caused by role conflict (Xu, 2019). The dynamic process of role conflict experienced by Palestinian teachers in Israel was also explored (Makkawi, 2002). Similarly, a survey of teachers at Gomal University found that role conflict had a negative correlation effect on job satisfaction (Khan, 2012). Japanese university teachers were the subjects of a study that used the Buss-Perry Aggression (BAQ) Questionnaire. The survey indicated that in the workplace, the higher the BAQ score on the Anger, Hostility, and Physical Aggression scales, the higher the level of role conflict (Kanchika et al., 2015). Another study found that because physical education teachers in United States schools assume both teaching and coaching roles, they may face role conflict that leads to job stress (Konukman et al., 2010). A study of 246 academic entrepreneurs also showed that academic identity was positively related to role conflict and that role conflict was negatively related to academic entrepreneurial performance (Zou et al., 2019). Based on these findings, we realize that role conflict is an important psychological factor affecting individuals in organizations.

### Academic Entrepreneurial Intent

Academic entrepreneurship is an innovative combination of risks and resources. First, it includes an element of risk because successful outcomes are not guaranteed. Second, it involves an organizational effort because it creates a new way of exploiting opportunities. Third, it must be innovative because it cannot copy what is already fully available in the market (Shane, 2003). Philpott et al. (2011) has provided nine examples of entrepreneurial examples that cut across the traditional academic paradigm: institutional grant policy ethos (public research funding provision); industrial consulting; private research contracts; intellectual property protection of patents and licenses; university incubators for innovative entrepreneurial projects; publication of scholarly articles; accredited education (producing highly qualified graduates); training courses in certain professions; and the establishment of technology parks. Academic entrepreneurial intent refers to the individual’s perceptions or ideas about an activity at the university. It has been shown that intents are effective predictors of behavior, especially rare and unpredictable behavior. Entrepreneurial intents indicate that when individuals have strong mental preparation and plans for entrepreneurial behaviors, they will not easily forego the behavioral payoff (Krueger and Brazeal, 1994; Krueger et al., 2000).

A study that surveyed teachers in Spanish higher education institutions found that entrepreneurial personality and talent were the two determinants of entrepreneurial intent for male and female teachers (Miranda et al., 2017b). It also indicated that entrepreneurial personality was more important for men and that entrepreneurial talent was more important for women. Therefore, these results indicate that academic entrepreneurship training in universities is an effective approach to stimulating women’s entrepreneurial intent. Another study that examined the determinants and process characteristics involved in the creation of a spin-off business (a form of academic entrepreneurship) and investigated the mechanisms of forming individual academic entrepreneurial intent found that entrepreneurial self-efficacy and perceived role models were relevant to academic entrepreneurial intent, with entrepreneurial self-efficacy having the greatest impact (Prodan and Drnovsek, 2010). A significant association has also been found between the determinants of students’ entrepreneurial intent and academic and demographic variables in higher education institutions in Portugal (Rodrigues, 2017).

### Theory of Planned Behavior

**FIGURE 1:**
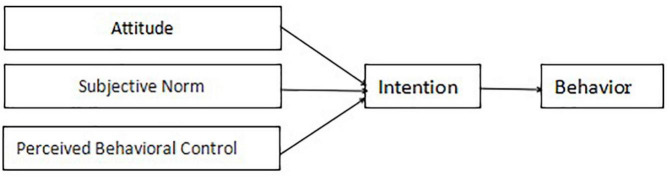
Theoretical model of planned behavior.

The theory of planned behavior suggests that the control of attitudes, subjective norms, and perceptual behaviors can accurately predict different types of behavioral intent and thus influence behavior formation (Ajzen, 1991). Attitude refers to the individual’s continuous positive or negative evaluation of a particular situation. Subjective norm refers to the individual’s perceived pressure on the surrounding environment as a result of adopting a particular behavior. Perceived behavioral control means that the stronger the resources and opportunities available to the individual and the fewer the expected obstacles, the stronger the intent to form a certain behavior. All three of these variables are influenced by belief. The theoretical framework of planned behavior is depicted in the figure below (see Figure 1).

## Hypotheses

An existing study surveyed 1,178 academics in Spanish universities with entrepreneurial attitude in academic entrepreneurship as a prior variable. It was an empirical study of entrepreneurial intent based on the theory of planned behavior, and it verified the proposed prior variable of entrepreneurial intent by constructing a structural equation model. Entrepreneurial intent was also found to be influenced by creativity, perceived utility, and entrepreneurial experience (Miranda et al., 2017a). A conceptual model was developed and validated based on the theory of planned behavior, integrating economic and psychological factors with human and social capital as economic factors and entrepreneurial attitudes, norms, and perceived control as psychological factors. It examined the entrepreneurial intents and actual innovative behaviors of academic scientists (*n* = 496) in Germany and found that psychological factors predicted entrepreneurial intent and that economic factors indirectly influenced entrepreneurial intent through psychological factors (Goethner et al., 2012). Similarly, a qualitative study based on grounded theory and the theory of planned behavior was conducted to construct a model for the factors of academic entrepreneurship among teachers in Chinese research universities. Human capital and role conflict were used as prior variables, and the literature on theory was used to verify the impact on academic entrepreneurial intent (Su and Zhao, 2017). The results showed that entrepreneurial spirit is a key factor in the formation of academic entrepreneurial intent among teachers in higher education institutions.

Previous literature has thus demonstrated that university environment is a key factor in the formation of psychological capital and role conflict for teachers. Psychological capital and role conflict are also effective in motivating teachers to be more productive and satisfied with their work and resist work-related stress. Therefore, we propose the following hypotheses:

H1: Psychological capital has a positive impact on attitudes toward academic entrepreneurship.

H2: Psychological capital has a positive influence on perceptual behavioral control.

H3: Role conflict has a negative effect on perceptual behavioral control.

In the empirical literature on the influence of academic attitudes and perceptual behavioral control on academic entrepreneurial intent, most studies have focused on student populations. Literature on teachers’ attitudes, perceptual behavioral control, and academic entrepreneurial intent is relatively sparse (Ferreira et al., 2012; Nguyen et al., 2019; Mahfud et al., 2020). However, according to the generality of predicting human behavior in the theory of planned behavior proposed by Ajzen (2011), attitudes and perceptual control behavior influence individual intents. Positive academic attitudes and perceptual control are thus effective in predicting scholars’ entrepreneurial intent (Schlaegel and Koenig, 2014; Fernandez-Perez et al., 2015). Therefore, hypotheses H4 and H5 of this study are proposed.

H4: Academic entrepreneurial attitudes have a positive impact on academic entrepreneurial intent.

H5: Perceptual behavioral control has a positive impact on academic entrepreneurial intent.

**FIGURE 2:**
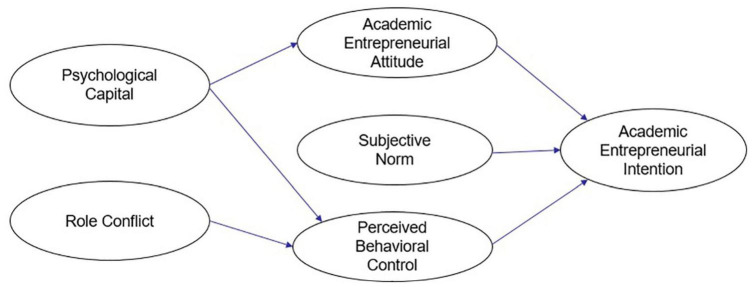
Research framework.

According to Ajzen (1991), the prior independent variable of attitudes, perceptual control, and subjective norms is believed to be belief, which is not an innate trait but rather formed by an individual’s exposure to the social environment. Goethner et al. (2012); Huyghe and Knockaert (2015), Foo et al. (2016), and Urban (2019) found that the process of forming academic entrepreneurial intent is often influenced by an individual’s surroundings, friends, colleagues, and role models. Academic entrepreneurship is characterized by an intrinsic orientation within individuals to strive for social distinction rather than social norms. Contextual factors indirectly predict entrepreneurial intent through attitudes and perceptual control rather than social norms (Krueger et al., 2000). Therefore, hypothesis H6 of this study is proposed (see Figure 2).

H6: Subjective norms have a positive impact on academic entrepreneurial intent.

## Methodology

The teachers from 17 universities in southern China are selected as research objects. A total of 557 questionnaires were sent out and recovered between May 7th 2021 and June 22nd 2021. At this point, SPSS 22.0 was used to check the data, which included 32 invalid questionnaires. In the end, 525 valid questionnaires were collected with an actual recovery rate of 94%. Descriptive statistical analysis was conducted on the basic data of participants (see Table 1).

**TABLE 1:** Descriptive statistics (*N* = 525).

Variable	Project	Frequency	Percentage%
Gender	Male Female	310 215	59% 41%
Age	25–35 36–46 47–57 58 or above	158 234 76 57	30% 45% 14% 11%
Education	Junior College Education Bachelor’s Degree Master’s Degree Doctoral Degree	45 134 177 169	8% 26% 34% 32%
Major	Science and Engineering Social Science Medical Science Art	342 125 36 22	65% 24% 7% 4%
Form of Academic Entrepreneurship	The task of part-time universities and research institutes for academic entrepreneurship. Full time academic entrepreneurship No academic entrepreneurship	434 19 72	83% 3% 14%

## Measures

In the present study, data was collected through the questionnaire method. Participants had to respond to a series of statements using a Likert’s five-level scale (“totally agree,” “agree,” “general,” “disagree,” and “totally disagree”)The distribution is recorded as 5, 4, 3, 2, and 1, and the total score of each respondent’s attitude is the sum of the scores gained from the answers to each item in the questionnaire. The total value can be said to be the attitude of each respondent or the different states on each scale.

The scale of psychological capital reference from Luthans and Youssef-Morgan (2017) four scale items of efficiency, hope, optimism and resilience. The scale of role conflict referenced Bartunek and Rynes’s (2014) three scale items, which include time conflict, problem conflict, and knowledge conflict. The scale of academic entrepreneurial attitude and perceived behavior referenced Liñán and Chen’s (2009) three scale items. The scale of subjective norm referenced three of Bercovitz and Feldman’s (2008) seven scale items. The scale of academic entrepreneurial intention referenced Liñán and Chen (2009) and Obschonka et al. (2015).

## Analysis and Results

In this study, reliability analysis, discriminant validity and convergence validity analysis, and correlation analysis were conducted on the collected valid sample data of *N* = 525, and a structural equation model was constructed. The results obtained are shown in Tables 2–6.

**TABLE 2:** Construct validity.

Item	1	2	3	4	5	6
PC4	**0.849**	0.063	0.091	−0.009	0.102	0.034
PC3	**0.810**	0.068	0.129	−0.050	0.071	0.118
PC2	**0.786**	0.069	0.062	−0.052	0.058	0.186
PC1	**0.772**	0.055	0.095	−0.121	0.120	0.206
SN3	0.065	**0.877**	0.164	−0.054	0.083	0.058
SN2	0.088	**0.876**	0.081	−0.065	0.103	0.072
SN1	0.078	**0.850**	0.164	−0.055	0.092	0.106
AEI1	0.109	0.162	**0.890**	−0.073	0.150	0.049
AEI2	0.126	0.146	**0.879**	−0.105	0.195	0.116
AEI3	0.168	0.168	**0.767**	−0.124	0.188	0.280
RC1	−0.061	−0.034	−0.095	**0.862**	−0.019	−0.122
RC2	−0.069	−0.074	−0.063	**0.860**	−0.064	−0.121
RC3	−0.064	−0.059	−0.087	**0.851**	−0.030	−0.138
AEA2	0.152	0.112	0.100	−0.022	**0.838**	0.153
AEA3	0.087	0.116	0.189	−0.053	**0.831**	0.200
AEA1	0.091	0.068	0.196	−0.047	**0.789**	0.174
PBC2	0.155	0.059	0.138	−0.121	0.203	**0.789**
PBC3	0.197	0.109	0.171	−0.124	0.162	**0.767**
PBC1	0.187	0.089	0.071	−0.203	0.189	**0.744**

*Factor load >0.5 are indicated in bold.*

**TABLE 3:** Analysis results of reliability and convergent validity.

Variable	Item	*S.E.*	*t*-value	*p*	CR	AVE	α
PC	pc1				0.851	0.588	0.850
	pc2	0.059	16.189	***			
	pc3	0.058	16.974	***			
	pc4	0.058	17.156	***			
RC	rc1				0.849	0.652	0.848
	rc2	0.055	18.262	***			
	rc3	0.056	18.105	***			
SN	sn1				0.872	0.694	0.871
	sn2	0.055	20.164	***			
	sn3	0.055	20.651	***			
AEA	aea1				0.837	0.633	0.833
	aea2	0.061	16.646	***			
	aea3	0.060	17.399	***			
PBC	pbc1				0.791	0.558	0.790
	pbc2	0.068	14.829	***			
	pbc3	0.070	14.789	***			
AEI	aei1				0.896	0.743	0.893
	aei2	0.040	26.587	***			
	aei3	0.042	22.245	***			

***p < 0.001, **p < 0.01, and *p < 0.05.

**TABLE 4:** Correlation analysis.

	1	2	3	4	5	6
1.PC	**0.767**					
2.RC	−0.184**	**0.807**				
3.SN	0.205**	−0.163**	**0.833**			
4.AEA	0.290**	−0.150**	0.268**	**0.796**		
5.PBC	0.418**	−0.354**	0.255**	0.467**	**0.747**	
6.AEI	0.317**	−0.249**	0.366**	0.435**	0.401**	**0.862**

*P < 0.05, **P < 0.01, the diagonal is the square root of AVE.

**TABLE 5:** Model index.

Statistical test		Stander range	Results
Absolute fitness indicator	x^2^/*df*	<3	2.184
	RMSEA	<0.08	0.048
	GFI	>0.9	0.939
	AGFI	>0.9	0.919
Value-added fitness indicator	NFI	>0.9	0.939
	CFI	>0.9	0.966
	TLI	>0.9	0.959
	IFI	>0.9	0.966

**TABLE 6:** Hypothesis testing.

Path		Standardization coefficient	*S.E.*	*t*-value	*p*	*Conclusion*
H1	PC→AEA	0.386	0.053	7.260	***	Supported
H2	PC→PBC	0.467	0.051	8.797	***	Supported
H3	RC→PBC	−0.334	0.046	−6.690	***	Supported
H4	AEA→AEI	0.312	0.055	6.576	***	Supported
H5	PBC→AEI	0.242	0.058	5.000	***	Supported
H6	SN→AEI	0.261	0.051	5.772	***	Supported

***p < 0.001, **p < 0.01, and *p < 0.05.

As shown in Table 2, after the factor extraction of 19 items, six factors were finally extracted. The six factors that were extracted included PC (Psychological Capital), SN (Subjective Norm), AEI (Academic Entrepreneurial Intention), RC (Role Conflict), AEA (Academic Entrepreneurial Attitude), and PBC (Perceived Behavioral Control), which were consistent with the theoretical assumption of scale structure. The factor load was above 0.6, and the factor compliance was good. The questionnaire thus had good construction validity.

As depicted in Table 3, Cronbach’s Alpha value of PC, RC, SN, AEA, PBC, and AEI is above 0.7, CR value is above 0.7, and AVE value is above 0.5. According to Hair et al. (2010), in validity evaluation, the absolute value of estimated factor load should be above 0.5 at least. The optimal index value is above 0.7, and the average variance withdrawal (AVE) index value should be above 0.5. This questionnaire thus has good convergence validity.

The method proposed by Fornell and Larcker (1981) was also adopted to determine whether the convergence validity existed if the square root of AVE was higher than the correlation coefficient between the two variables. As shown in Table 4, the AVE square roots of PC, RC, SN, AEA, PBC and AEI were 0.767, 0.807, 0.833, 0.796, 0.747, and 0.862, respectively, which were all greater than their corresponding correlation coefficients, indicating that the questionnaire had good convergence validity.

As illustrated in Table 4, AEA was significantly positively correlated with PC (*r* = 0.290, *P* < 0.01). PBC was positively correlated with PC (*R* = 0.418, *P* < 0.01) and negatively correlated with RC (*r* = −0.354, *P* < 0.01). AEI was significantly positively correlated with SN (*r* = 0.366, *P* < 0.01), AEA (*R* = 0.435, *P* < 0.01), PBC (*r* = 0.401, *P* < 0.01). Therefore, the hypothesis is tentatively supported.

**FIGURE 3:**
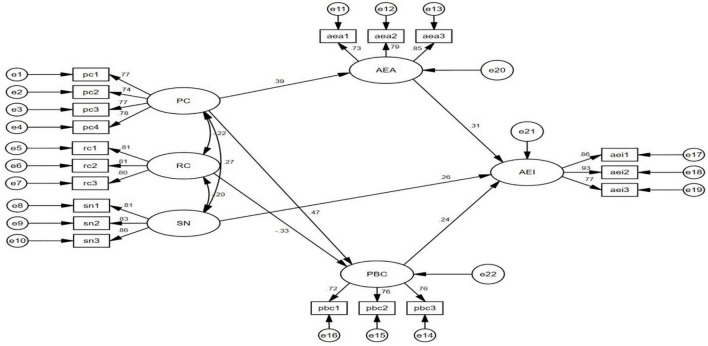
Structural equation model.

According to the test method of Bagozzi and Yi (1988), from Table 5 that x2/DF value is 2.184, RMSEA value is 0.048, GFI value is 0.939, AGIF value is 0.919, NFI value is 0.939 and CFI value is 0.966 (see Figure 3). The value of TLI was 0.959, and the value of IFI was 0.966. The fitting indexes of the model both reached ideal values, indicating that the fit of the structural equation model was good.

As shown in Table 6, PC had a significant positive effect on AEA (β = 0.386, *P* < 0.001). PC also had a significant positive effect on PBC (β = 0.467, *P* < 0.001), RC had a significant negative effect on PBC (β = −0.334, *P* < 0.001), and PC had a significant positive effect on AEA (β = 0.386, *P* < 0.001). AEA had a significant positive effect on AEI (β = 0.312, *P* < 0.001), PBC had a significant positive effect on AEI (β = 0.242, *P* < 0.001), and SN had a significant positive effect on AEI (β = 0.261, *P* < 0.001). Therefore, all six of the hypotheses in this study were supported.

## Discussion and Conclusion

Literature on academic entrepreneurship has neglected to investigate the entrepreneurial intent of teachers in higher education from a psychological perspective. Studies that use qualitative analysis are sparse, and most were conducted on university students (Baluku et al., 2020; Mahfud et al., 2020; Shi et al., 2020; Zhao et al., 2020). Used a qualitative method and discussed the link between the lack of psychological capital and teacher motivation. They argued that superior psychological capital will increase motivation, and that more research should be conducted that focuses on psychological capital and teachers (Viseu et al., 2016). Others have also argued that the roles of academic researcher and entrepreneur should be conceptually distinct in the process of academic entrepreneurship to avoid the negative effects of role conflict on academic entrepreneurship (Wood, 2011).

Based on these findings, the research model adopted in this study was constructed based on the theory of planned behavior. As shown in Table 6, the influence of academic attitudes, perceptual behavioral control, and subjective norms on academic entrepreneurial intent (H4, H5, and H6) were all supported. The results about the impact of academic attitude and perceptual behavioral control are in agreement with the findings of Goethner et al. (2012) and Miranda et al. (2017a). However, the effects of subjective norms on academic entrepreneurial intent did not hold in previous studies. Our results concerning the hypothesis of subjective norms are not the same as those of western scholars, but there are some relevant studies showing that the influence of subjective norms on academic entrepreneurial intent is supported when the sample is drawn from Chinese teachers in higher education (Su and Zhao, 2017; Shi et al., 2020). Social identity theory holds that subjective norms on academic entrepreneurial intent are established because they are shaped by the moderating effect of group identity (Terry et al., 1999). Therefore, we believe that this difference is due to variations in cultural environments. In a university context, in the absence of sociocultural and traditional entrepreneurship, academic entrepreneurial intent depends more on individual personalities than on subjective norms (de Janasz et al., 2007). The academic entrepreneurial intent of teachers in higher education is strongly supported by surrounding groups and environmental policies. When higher education administrators have participated in academic entrepreneurship, in particular, the possibility of academic entrepreneurship of the teachers in higher education is improved on the whole (Brennan et al., 2005; Su and Zhao, 2017).

This study also supported the hypotheses that psychological capital on the control of attitudes and perceived behaviors have an effect on academic entrepreneurship (see Table 6). We found that although there are few directly relevant prior studies, there is indirect evidence that psychological capital exists as a significant predictor of academic entrepreneurial intents. As the first aim of the study demonstrated, the more significant academic attitudes and perceptual behavioral controls are, the greater the predictability of academic entrepreneurial intents will be. Because academic attitude and perceptual behavioral control are significantly influenced by psychological capital, a study conducted by Contreras et al. (2017) on the relationship between psychological capital and entrepreneurial intents found that entrepreneurial intents are correlated with the dimensions of psychological capital. It has been shown that a positive effect exists in the interaction between psychological capital and entrepreneurial intent (Yalap et al., 2020) and that psychological capital has a significant impact on entrepreneurial intents through traditional financial, human, and social capital (Zhao et al., 2020). Both startup capital and psychological capital are important predictors of entrepreneurial success, but psychological capital is a better predictor (Baluku et al., 2016). Therefore, we argue that when psychological capital controls for academic entrepreneurial attitudes and perceived behaviors are supported by hypothesis testing, the latter (PC) exerts a stronger influence than the former (AEA and PBC) in predicting the indirect role of academic entrepreneurial intents. Concurrently, based on the premise that the research object is Chinese teachers in higher education, we also found that the psychological capital of Chinese teachers in higher education has an important effect on job performance, scientific research, and innovation behavior and job engagement (Juan, 2021). Academic entrepreneurship is one of the ways of scientific research innovation, but there is still a scarcity of empirical literature studies on this topic in China. This study used empirical research methods to prove that the psychological capital of Chinese teachers in higher education is one of the important factors that predict academic entrepreneurship.

We also found that our hypothesis about the effect of role conflict on the control of perceptual behavior (H3) is supported. This conclusion is the same as the one obtained by Su and Zhao (2017), who performed a qualitative analysis based on a model of teacher academic entrepreneurship constructed in a Chinese context.

With respect to spin-off business creation (a form of academic entrepreneurship), the conflict between entrepreneurship and social identity remains common (O’kane et al., 2015; Wang et al., 2021). Research shown that role conflict is negatively related to the performance of academic entrepreneurship, as explained by social identity theory (Zou et al., 2019). To this end, based on the properties of the theory of planned behavior (where human control over will and behavior is considered as a continuum), we explain how enhancing the academic entrepreneurial intent of teachers in higher education can be achieved by increasing their perceived behavioral control (behavior under non-volitional control), thereby reducing the negative impact of role conflict. Based on social learning theory, it has been demonstrated that the more experience academic entrepreneurs have, the weaker the negative impact of role conflict will be (Zhang et al., 2021). As this study confirms, perceptual behavioral control effectively reduces the negative effect of role conflict on the academic entrepreneurial intention of teachers in higher education.

In summary, this study showed that psychological capital is an important leading indicator that can predict academic entrepreneurial intents, and in the context of colleges and universities, managers can reduce the negative impact of role conflict caused by teachers who serve as entrepreneurs and academics through perceptual behavioral control. As Peng (2018) has said, “PASSAGE.” Dealing with the relationship between difference and legacy is thus key to achieving academic entrepreneurship. We hope that in comparing our findings with research that has been conducted in other countries, the universal implications of our research have been made clear and can provide useful suggestions about academic entrepreneurship for administrators in universities in China and beyond.

## Limitations

The limitations of this study are twofold. First, our sample was selected from a group of university teachers living in the southern region of China, and the geographical differences may cause the findings of the study to differ. In order to address this issue, future research could take the form of a comparative study of university teachers in China and Korea. Second, designing survey questions to assess teachers’ entrepreneurial intent is a challenging task. Open-ended questions can be problematic in terms of quality data recovery and the analysis of results. Future research could use textual information mining and data analysis to obtain key terms from teacher postings and comments about academic entrepreneurship on the Internet. Third, the influencing factors of the academic entrepreneurial intents of teachers in higher education are complex and diverse. In the future, we can introduce regulatory variables or intermediary variables to further explore academic entrepreneurial intent.

## Data Availability Statement

The original contributions presented in the study are included in the article/Supplementary Material, further inquiries can be directed to the corresponding author/s.

## Ethics Statement

Written informed consent was obtained from the individual(s) for the publication of any potentially identifiable images or data included in this article.

## Author Contributions

All authors listed have made a substantial, direct, and intellectual contribution to the work, and approved it for publication.

## Conflict of Interest

The authors declare that the research was conducted in the absence of any commercial or financial relationships that could be construed as a potential conflict of interest.

## Publisher’s Note

All claims expressed in this article are solely those of the authors and do not necessarily represent those of their affiliated organizations, or those of the publisher, the editors and the reviewers. Any product that may be evaluated in this article, or claim that may be made by its manufacturer, is not guaranteed or endorsed by the publisher.
